# Stakeholder views and attitudes towards prenatal and postnatal transplantation of fetal mesenchymal stem cells to treat Osteogenesis Imperfecta

**DOI:** 10.1038/s41431-019-0387-4

**Published:** 2019-03-27

**Authors:** Melissa Hill, Celine Lewis, Megan Riddington, Belinda Crowe, Catherine DeVile, Anna L. David, Oliver Semler, Magnus Westgren, Cecilia Götherström, Lyn S Chitty

**Affiliations:** 1 North East Thames Regional Genetics Service, Great Ormond Street Hospital for Children NHS Foundation Trust, London, UK; 2 Genetics and Genomic Medicine, UCL Great Ormond Street Institute of Child Health, London, UK; 3 Osteogenesis Imperfecta Service, Great Ormond Street Hospital for Children NHS Foundation Trust, London, UK; 4 Institute for Women’s Health, University College London, London, UK; 5 Faculty of Medicine and University Hospital Cologne, Department of Pediatrics, University of Cologne, Cologne, Germany; 6 Department of Clinical Science, Intervention & Technology, Karolinska Institutet, Stockholm, Sweden

**Keywords:** Psychology, Metabolic disorders

## Abstract

The Boost Brittle Bones Before Birth (BOOSTB4) clinical trial is investigating the safety and efficacy of transplanting fetal derived mesenchymal stromal cells (MSCs) prenatally and/or in early postnatal life to treat severe Osteogenesis Imperfecta (OI). This study aimed to explore stakeholder views to understand perceived benefits or concerns, identify ethical issues and establish protocols for support and counselling. Semi-structured qualitative interviews were conducted with three groups; 1. Adults affected with OI, with and without children, and parents of children affected with OI; 2. Health professionals who work with patients with OI; 3. Patient advocates from relevant patient support groups. Interviews were digitally recorded, transcribed verbatim and analysed using thematic analysis. Interviews with 56 participants revealed generally positive views towards using fetal MSC transplantation to treat OI. Early treatment was considered advantageous for preventing fractures and reducing severity and could bring psychological benefits for parents. Common concerns were procedure safety, short/long-term side effects and whether transplantation would be effective. Difficulties inherent in decision-making were frequently discussed, as treatment efficacy is unknown and, by necessity, parents will make decisions at a time when they are vulnerable. Support needs may differ where there is a family history of OI compared to an unexpected diagnosis of OI. Explaining fetal MSC transplantation in a way that all parents can understand, clear expectation setting, psychological support and time for reflection during the decision-making process will be crucial to allow parents to make informed decisions about participation in the BOOSTB4 clinical trial.

## Introduction

Osteogenesis Imperfecta (OI) is a rare metabolic bone disorder that affects ~1 in 20,000 births [1–3]. Common features include bone fragility, osteopenia, short stature, atypical skeletal development, brittle teeth, hearing loss and hypermobile joints [1, 2]. Variants in the *COL1A1* and *COL1A2* genes that either reduce the amount of collagen or impact on collagen structure are causal in 85–90% of cases [4]. OI has been classified as mild (type 1), moderate (type 4), severe (type 3) or lethal (type 2) [5]. OI is a heterogeneous condition and severity varies widely, including between family members with the same genetic variant [6]. Severe forms can sometimes be identified before birth when ultrasound detects fractures, reduced fetal growth or bowing of the limbs.

There are no curative interventions for OI. Treatment options focus on reducing fractures and deformity, providing relief from pain and improving mobility and day-to-day function [6]. Bisphosphonates are often offered when children have moderate to severe OI as they increase bone mineral density and improve bone strength and, as a result, reduce fracture risk and bone pain [7]. Surgery, such as intramedullary rodding, maintains bony alignment and reduces fracture risks when long bones are bowed or if fractures are recurrent [6]. Physical and occupational therapy are also commonly used to help with mobility and function [8].

Transplantation of fetal mesenchymal stem cells (MSCs) is a new approach to treating OI that, although it will not cure the condition, does have the potential to modify severity [9, 10]. As OI can cause damage early in fetal life, prenatal or early post-natal (first year of life) stem cell transplantation (SCT) could help to ameliorate the disease process at a time of rapid skeletal development and before additional pathology occurs as a result of fractures. To date clinical experience with SCT for OI is limited, and further studies are needed [10, 11]. The Boost Brittle Bones Before Birth (BOOSTB4) Phase I/II clinical trial will examine the safety and, efficacy of SCT for severe OI. The aim of our study was to explore the views and attitudes of key stakeholders towards SCT and the proposed BOOSTB4 clinical trial.

## Materials and methods

A qualitative approach using one-to-one semi-structured interviews was used to enable an in-depth exploration of stakeholder views. NHS Research Ethics Committee approval was obtained (16/NS/0084).

### Sampling and recruitment

Three participant groups were recruited; (1) Adults affected with OI, with and without children affected with OI, and parents of children affected with OI, who were not affected with OI themselves (“patients and parents”); (2) health professionals; and (3) patient advocates from relevant support groups. Patients and parents were recruited through four paediatric (three in England one in Scotland) and one adult OI specialist service (in England). Potential participants who met the inclusion criteria of being 16 years and over and able to communicate in English were identified by the clinical team. The participant information was either sent by mail to potential participants or provided at clinic appointments. An advertisement was placed on the Brittle Bone Society (UK patient support group for families with OI) website and Facebook page. Potential participants were asked to contact MH if interested in participating or, if attending a clinic, they could complete a “consent to contact” form and MH then contacted them by email or telephone. Health professionals and patient advocates were identified by the research team with input from the OI specialist services and invited to participate by MH via email.

### Information provided about fetal MSC transplantation and the BOOSTB4 clinical trial

A BOOSTB4 Fact Sheet, developed by MH and reviewed by the multi-disciplinary research team, included the following key information about the planned BOOSTB4 clinical trial; 1. The trial will look at the safety and efficacy of SCT initiated during pregnancy or shortly after birth. 2. Early treatment was chosen to support engraftment, minimise risks of immunosuppression and because OI can cause damage very early in life. 3. Trial eligibility will be limited to severe forms of OI resulting from specific genetic variants. 4. Prenatal SCT has a 1% risk of miscarriage or pre-term birth. 5. SCT will be given several times over two years. 6. Fetal MSCs are obtained when women choosing to have a termination of pregnancy for personal reasons in the first trimester agree to donate fetal cells for research. 7. The number of children with OI previously treated with SCT is very small.

### Interviews

Semi-structured interviews were conducted face-to-face or via telephone by MH. Interviews were designed to last 30–40 min. Demographic questions included age, education level, ethnicity and number of children. The interview guide explored four topic areas; 1. Experiences of living with OI or working with families affected by OI. 2. Views on current treatments for OI. 3. Views on using SCT to treat OI. 4. Support and information needs for decision making. Findings from the first two topic areas will be reported separately.

### Data analysis

Interviews were digitally recorded, transcribed verbatim and anonymised. Pseudonyms were assigned to each participant. Data were analysed by MH and CL using the principles of thematic analysis [12]. MH and CL are experienced qualitative researchers with no previous research experience in OI, fetal therapy or SCT, which allowed a critical distance from the research topic. NVivo10 (QSR International, Pty Ltd, Australia) was used to facilitate analysis. Transcripts were coded into meaningful units of text. Codes were then grouped into broader categories and themes that were progressively reviewed and redefined. The three participant groups were initially treated as separate data sets. Emerging themes were then compared and ultimately combined. MH coded all transcripts and CL coded a sub-set (10%) of transcripts independently to ensure inter-rater reliability. No substantial differences in interpretation occurred.

## Results

Interviews were conducted with 56 participants; 25 patients and parents, two patient advocates and 29 health professionals. Nine patients and parents were recruited through the Brittle Bone Society and 16 through OI specialist services. As recruitment combined invitations and advertisements, a response rate could not be calculated. Two patient advocates were invited to participate, and both accepted. Of 43 health professionals invited to participate, 29 agreed (response rate: 67%). One health professional is a member of the BOOSTB4 research team. Interviews were conducted between 30/09/2016 and 15/02/2018, 19 in person and 36 by phone. Interviews lasted between 15 and 56 min (median = 30 min). Participant characteristics are described in Tables 1 and 2. Three overarching themes arose from the interviews; “potential benefits”, “concerns” and “decision making”.

**Table 1:** Health professional and patient advocate demographic data

	Total (*n* = 31)
Age group (years)	
21–30	2
31–40	8
41–50	10
51–60	11
Gender	
Female	23
Male	8
Profession	
Clinical geneticist	3
Fetal medicine specialist	3
Genetic counsellor	2
Nurse specialist (paediatric care)	3
Nurse specialist (adolescent/adult care)	1
Occupational therapist (paediatric care)	2
Endocrinologist / metabolic bone specialist (paediatric care)	7
Patient advocate	2
Physiotherapist (paediatric care)	5
Psychologist (paediatric care)	1
Rheumatologist (adolescent/adult)	2
Years working with families affected with OI	
≤5	13
6–15	11
16–25	7
Current practice location	
England (North)	4
England (Midlands)	5
England (London)	12
England (South)	8
Scotland	2

**Table 2:** Patient and parents demographic data

	Total (*n* = 25)
Affected with OI	
Yes	17
No	8
Gender	
Female	21
Male	4
Age group (years)	
17–24	4
25–35	9
36–45	8
46–55	2
>55	2
Ethnicity	
Asian British	3
Mixed	2
White British	20
Highest qualification	
High school	5
Degree or equivalent	20
Number of children	
None	8
Two	10
Three	6
Four	1
Number of children with OI	
None	9
One child with OI	10
Two children with OI	5
Three children with OI	1
Self-described OI type of self or child	
Type 1 or Mild	16
Type 4 or Moderate	1
Type 3 or Severe	7
Recruited from	
Brittle Bone Society	9
OI specialist service	16

### Potential benefits

#### Modifying the severity of OI will have clinical and psychosocial benefits

Participants spoke about OI as a condition that impacts on all areas of life and felt that if SCT was successful the result would be wide-ranging clinical and psychosocial benefits for the child and their family. The potential clinical benefits most commonly highlighted were; a reduction in the number of fractures, fewer bone deformities and improved growth. There was also hope that SCT could benefit chronic pain, tiredness and dental problems. Participants felt that clinical improvements would lead to multiple psychosocial benefits for children as fewer hospital visits for fractures and a reduced need for surgery would mean more time at school and greater involvement in family and social activities (Table 3: Quote 1). In addition, improvements in function would allow greater independence as children would be “less dependent on wheelchairs or other people”. Many commented that quality of life would be improved and that SCT could help the child to be “stronger and healthier and happier” and have a more “normal life.”

**Table 3:** Participant quotations to support themes

Quote Number	Quotation
1	“They’re not going to need to spend as much time in hospitals or sort of in casts and things like that… It would have big implications for families being able to access more schooling and more social time.” *Luke, health professional from an OI specialist team*
2	“…if there was a treatment that reduced a risk of fractures and then, then we would certainly be open to looking at it and considering it. Because I think what’s, what’s really hard about OI is, is that constant fear of fracturing” *Carmel, parent of a child with OI*
3	“I think it would have a massive positive effect on bonding, development, everything else if you just knew that the chances of them fracturing was going to be less, I think you’d feel a lot more robust about it.” *Sally, health professional from an OI specialist team*
4	“With the more severe types, obviously as soon as they start to move around in the womb they fracture so anything that could reduce this risk is good.” *Mary, adult with OI, who has a child with OI*
5	“You feel entirely powerless with a genetic issue. So it would be amazing to be able to have and feel like you could influence or to improve, improve the situation” *Annie, parent of a child with OI*
6	“I was quite overjoyed. I think when I first started reading about [SCT] because I was thinking instantly about my family, my future family and how that would be hugely beneficial.” *Nathan, adult with OI*
7	“…if I did have a child that has multiple breaks and I have got the condition myself it would be very difficult for me on a practical side moving a child that’s got lots of breaks.” *Mia, adult with OI*
8	“I think it’s really exciting that, you know, something new for the OI community and yeah could potentially be really life changing for people” *Lily, adult with OI*
9	“I think you’d probably ask whether you could introduce any other diseases or other conditions through doing that” *Sam, parent of a child with OI*
10	“I would say after pregnancy just purely because of the risk of miscarriage” *Jill, adult with OI*
11	“Obviously there’s a risk that there’ll be no difference or things might not go, you know, they may even get worse.” *Ben, health professional from an OI specialist team*
12	“How many children have had it already? How much has this been tested? Because I think you’d have to be very brave to, you know, be one of the first” *Olivia, adult with OI who has a child with OI*
13	“Stem cells are in the paper all the time, ‘magic new therapy’. And again I do worry a little bit about some families which will go ahead and say ‘yes, yes’ and think that possibly, the outcome may be different than what it will actually be.” *Julie, health professional from an OI specialist team*
14	“I think going through the process of treatment and then not having the outcome you wanted would be really, really difficult.” *Laura, adult with OI*
15	“We want to do everything we can and to help to make sure that they’re making an informed decision that isn’t just a, sort of, reaction to the situation that they’re in, is really challenging because you can say, yeah, that the acute grief will make them quite vulnerable to maybe making a decision that they wouldn’t make in a different setting” *Emma, genetic counsellor*
16	“If I put myself in the shoes of when we were pregnant… if I didn’t have an OI child I think I might be reluctant to do something, I’d be honest, but if I had an OI child my God I’d be wanting to try everything… And I think it will be quite hard for someone who doesn’t fully understand what OI means.” *Alex, parent of a child with OI*
17	“I would have had the treatment bar the risks, bar wherever the stem cells are from or I would have given it a go and I would have had to because I couldn’t in six months’ time be sitting by that baby in the hospital with two cracked femurs because how could I then say to myself I did everything I could for you because I didn’t take that risk.” *Ameenah, adult with OI who has a child with OI*

Possible psychosocial benefits for parents were frequently discussed. Some participants noted that reducing the likelihood of fracturing would decrease the emotional toll of OI as there wouldn’t be the same fear of fractures and distress at seeing the child in pain (Table 3: Quote 2). In addition, some participants suggested that if SCT led parents to view their child as less fragile then they would have greater confidence when handling their child and that this could have a positive impact on bonding (Table 3: Quote 3).

#### Early interventions are viewed positively

Participants valued that treatment could begin early in life. As fractures can occur *in utero*, during the birth process or soon afterwards, it was viewed as being particularly beneficial to intervene during pregnancy (Table 3: Quote 4). Possible psychological benefits for parents included SCT being an additional option to consider at the time of diagnosis that would offer parents some “hope”. Starting treatment during pregnancy or shortly after birth may also alleviate some of the feelings of anxiety and powerlessness that can occur when OI is diagnosed (Table 3: Quote 5). Some health professionals also felt that offering SCT in pregnancy would be valued by parents who would not consider termination of pregnancy as “they could improve the quality of life” of the child.

#### Positive impacts on starting a family when you have moderate/severe OI yourself

Several adults affected with OI commented that they did not want to pass OI to their child. SCT was seen to offer an additional option to consider when starting a family that may allow having a child to be viewed more positively (Table 3: Quote 6). Some participants noted that it is difficult to care for a child with OI when you have OI yourself (Table 3: Quote 7) and it may be possible to take a more active role in parenting if SCT can reduce the severity of the child’s OI.

#### Reduce the need for bisphosphonates

Although most participants spoke positively about bisphosphonate treatment for children with OI and valued the observed improvements to fracture rates, pain and tiredness, they also noted that many children “struggle” with cannulation and the frequency of treatments can be “burdensome for families”. Consequently, SCT would be welcomed if improvements in bone strength meant that bisphosphonates could be given at less frequently or not be needed at all.

#### Research about OI is important

Participants were very positive that “innovative” research on new treatments for rare conditions such as OI were being undertaken (Table 3: Quote 8). Some health professionals commented that the evaluation of SCT as a treatment for OI may ultimately lead to similar therapies for skeletal dysplasias and other rare diseases which “could be a huge leap forward”.

### Concerns

#### The stem cells are fetal in origin

Some participants, from both the patients and parents and health professional groups, commented that they personally felt “uncomfortable” or “worried” about the fetal origin of the stem cells. Reasons included; not agreeing with termination of pregnancy, which was sometimes linked to the person’s religious beliefs, views around when a “person becomes a person”, concern that supporting the use of fetal cell therapies may encourage more terminations as “people would more easily choose abortion, thinking that [the stem cells] could be used for something good” and the fetus is unable to give consent. Some of these participants said that their concern about the origin of the cells was not “clear cut” or said that they were “slightly on the fence” as they could see that some good could come out of using the cells. The majority of participants reported not having any ethical concerns about the fetal origin of the stem cells and felt that if the mother had already made the decision to terminate and had given informed consent to donate the cells, then this was an “opportunity for something good to come out of a bad situation”. It was acknowledged that the fetal origin of the cells could be an issue for some people. One health professional noted that some parents may be “conflicted” as they have concerns about the origin of the cells, but also want their child to be well.

#### Possible risks to the child and mother

All participants wanted to be confident that the stem cells were as safe as possible for both child and mother and information about possible short- and long-term side effects of having SCT would be important in decision making. Some also commented that parents would want to be confident that the stem cells had been tested to prevent diseases being passed from the donor (Table 3: Quote 9).

The quoted 1% risk of miscarriage or pre-term birth associated with prenatal SCT was noted as a concern by most participants. For some in the patients and parents group this risk was viewed as unacceptable and they said that they would prefer to wait until after the baby was born to consider SCT (Table 3: Quote 10). For other participants the risk of miscarriage was discussed as a factor that would need to be carefully weighed against the possible benefits of initiating SCT during pregnancy.

#### The uncertainty associated with a very new treatment approach

The fact that SCT is a new approach for treating OI was discussed as a concern by some participants who highlighted the current uncertainties; SCT may not work as expected (Table 3: Quote 11), there may be side-effects or complications, including some that may not have been anticipated, and we do not know the long-term implications. Several participants commented that you would have to be brave to try a new treatment and some felt that parents may be anxious about having a new and unproven treatment (Table 3: Quote 12).

#### Potential for false hopes and disappointments

The potential for unrealistic expectations around the impact of SCT was raised as a concern by some participants (Table 3: Quote 13). It was also acknowledged that if the treatment did not work as expected that this would be “quite a big blow” and a “disappointment” after going through the process of deciding and then undergoing treatment (Table 3: Quote 14).

#### Research during pregnancy

Participants acknowledged that research during pregnancy needed to be “put together and very carefully, with careful protocols and handled sensitively”, particularly if there was an increased risk of miscarriage. However, no-one was opposed to conducting research in pregnancy and the importance of conducting research to “move new treatments and therapies forward” was noted. Many participants commented that if people were well informed of the risks and any limitations of the intervention that it should be up to the individual to choose whether to participate.

### Decision making

#### Parents face a difficult decision

Deciding whether to take part in the BOOSTB4 clinical trial was acknowledged by all participants to be a difficult decision that would be “considered differently by every family”. Many participants felt that deciding about SCT at the time of diagnosis would be particularly difficult as parents are made vulnerable by their shock and grief and it may be harder to absorb information at this time (Table 3: Quote 15). Some participants also noted the added responsibility of the decision because “a decision about someone else’s life is a big decision”. Another key issue was uncertainty around efficacy and long-term outcomes and the need to take a “leap of faith”. Decision making may also be difficult as it is hard to predict the impact of OI, even when other family members are affected. Many participants highlighted that previous experience may influence decision making and that weighing up risks and benefits may be more difficult for parents with no previous experience of OI (Table 3: Quote 16).

#### Decisions made during pregnancy or after the birth of the child

When discussing the issues specific to decision making in pregnancy, the risk of miscarriage was invariably thought to be a major consideration for parents. Other issues frequently discussed were the need to consider risks for both baby and mother and time pressures, as pregnancy brings “a time limit and that sense of urgency with making your decision”. Some health professionals and patient advocates also spoke about the added difficulty of weighing up termination of pregnancy when there was an offer of an intervention. They thought that some parents would opt for termination and “wouldn’t even consider [SCT] because for them they don’t want to take the risk of disability”, while other parents will be more undecided about termination and the offer of an intervention will add to the complexity of an already “emotionally laden” decision.

Many participants thought that decision making after the baby was born was likely to be “slightly easier” as there was not a procedural risk to the health of child and the time pressures of pregnancy are removed. They acknowledged, however, that the other complexities of the decision remained, and other factors may impact the decision. For example, some participants noted that when the baby is born, OI becomes much more real and you may be motivated to opt for SCT when the baby is in front of you and you are dealing with multiple fractures.

#### Potential pressure to choose SCT

Participants were asked if they thought parents might feel pressured to take part in the clinical trial. While it was acknowledged that parents may feel pressure from health professionals to participate, most felt that this would be very dependent on the health professional and their approach to explaining the clinical trial. Emotional pressures to accept the offer of SCT were discussed by some participants. For example, parents may place pressures on themselves to be “good” parents and do everything they can to help the child or they may be influenced by feelings of guilt for passing on the gene. Emotional pressures were evident when some participants in the patient and parent group described their willingness to “do anything” to reduce the severity of the condition (Table 3: Quote 17). Several participants commented that when the choice is to do nothing or to do something that could potentially help, it is very difficult to do nothing and that “it would be easy to think there’s only one option if you’re kind of given the option of helping your baby or not helping your baby”.

#### Practical considerations

Practical considerations raised included the need to travel to a specialist centre to have SCT and for some follow-up appointments. It was noted that it can be difficult travelling with a baby affected with OI and it can sometimes be difficult for adults with OI to travel long distances. Issues such as taking time off work to attend appointments and sorting out childcare for siblings were also noted. The fact that SCT would not be a one off intervention but may have to be performed every six months was not seen as a barrier as this was not dissimilar to treatment with bisphosphonates, with which many families are familiar.

#### Information and support needs for decision making

Participants felt that information in plain language, emotional support and time for reflection would make it possible for parents to make an informed decision. Supporting parents to make the best decision for their individual family was a priority for health professionals. Many participants highlighted the need for health professionals to be clear that the treatment may not work and felt that a key component of the discussion would be expectation setting. Several participants spoke about how substantial support would be needed for decision making and that counselling would be required to work through the emotional aspects of the decision and to “question and challenge” parental choices. Many also felt that it would be important to speak to a counsellor or nurse specialist who was independent of the trial.

Many participants highlighted that counselling and support needs may differ where there is a family history of OI compared to a new and unexpected OI diagnosis. Families with no previous experience of OI may need much more support and input from OI specialist teams and patient groups to allow them to get a realistic understanding of what OI would mean for their family. Partners of adults with OI may also need this additional support and information about the condition. In addition, some parents may want to speak to their own OI clinician with whom they have an existing relationship and have built up trust.

## Discussion

As new approaches to treatments are developed, stakeholder views must be considered to ensure acceptability and to explore information and support needs for potential participants of clinical trials and for future implementation. Most participants in our study reported feeling positive about SCT and the BOOSTB4 trial but also raised concerns about possible risks and the complexity of decision making. These views reflect those seen in other studies which report stakeholders holding strong, yet cautious, support for stem cell research [13]. Offering SCT during pregnancy was valued by participants as this would allow intervention before fractures occur. Accordingly, many participants in the patients and parents group said that they would consider SCT during pregnancy, but some felt that they would not be prepared to put the pregnancy at risk of miscarriage and would wait until after the child was born to decide.

The potential clinical and psychosocial benefits of SCT cited by participants, where fewer fractures and less time in hospital was anticipated to lead to more school and social time and a more normal life, reflect research findings exploring the experiences of children affected with OI. Two recent systematic reviews found that the clinical features of OI have a significant impact on quality of life [14] and many psychosocial implications such as feelings of isolation and being different [15]. Potential psychosocial benefits for parents were also discussed and participants thought that reducing OI severity would allow parents to view their child as more robust, alleviating fear of fractures and giving confidence for handling their baby. Other studies have shown that fear of fractures and the uncertainty of when the next fracture could occur are a constant feature of family life when a child has OI [16, 17], and parents face feelings of stress, helplessness, guilt, anxiety, depression and a lack of confidence in parenting skills [16, 18, 19]. The possibility of intervention at the time of diagnosis was anticipated to bring hope to parents and similar psychological benefits have been seen in studies looking at motivators for parents participating in paediatric research, for example parents considering research trials for Duchenne muscular dystrophy (DMD) reported feeling optimism and empowerment from potentially being able to impact the disease course [20, 21].

Several factors combine to make decision making complex for parents that could be offered SCT. Although participants felt strongly that decision making would be difficult, they also thought that it would be possible to support informed decision making. In a study looking at views of a clinical trial of maternal gene therapy for severe fetal growth restriction, women with a previous affected pregnancy also noted the difficulty of decision making at the time when a problem with the pregnancy was diagnosed [22]. In common with this study, it was felt that women would place themselves under emotional pressure to participate in a trial that would help their baby, but they felt that informed decisions would be possible with good information and careful counselling [22]. Parents invited to participate in research trials during pregnancy have previously described limitations in consent procedures and lack of information [23] and insufficient time for discussion with research staff [24]. In the paediatric research setting, parents of children invited to take part in research wanted more discussion time with researchers [20] and the importance of expectation setting has been highlighted as parents had not considered that the trial might fail [21]. Careful consideration is needed to develop study materials and counselling strategies. Suggestions for good practice are summarised in Table 4.

**Table 4:** Summary of good practice points when offering stem cell transplantation to parents

Good practice points
Significant support needed to help with decision making
Include both partners in the counselling
Allow time for discussion and time to go away and think
Provide good written information to take away
Provide carefully considered links to descriptions of OI
Diagnosis in pregnancy - explore the option to terminate and take care with the timing of the SCT offer
Describe the commitment needed (e.g., number of appointments, length of follow-up)
Be clear with expectation setting and be honest that outcomes are uncertain
Supply details of previous research, including numbers previously treated and case studies
Clear explanation of possible short and long-term side effects and unanticipated adverse events
Clear explanation of procedural risks for baby and mother
Clearly state that the stem cells are fetal in origin
Discuss other options for treatment
Careful framing of SCT to avoid pressure to participate
Explore and mitigate possible pressures parents may place on themselves to participate
Put families in touch with someone independent
Be aware that families faced with a new and unexpected diagnosis of OI may need much more input from the OI specialist health professionals and patient support groups

*SCT* stem cell transplantation

For parents with no previous experience of OI, dealing with a new and unexpected diagnosis of a rare condition will add to the difficulty of making a decision about participating in the BOOSTB4 clinical trial. In line with participant views on diagnosis in this study, other qualitative research with parents of children with OI shows that diagnosis can initially be a devastating experience and parents often feel that more support and information is needed at this time [18, 19]. Accordingly, the need for additional support and more detailed information about the condition for parents without previous experience of OI was noted by participants in this study. Additional support requirements from OI teams need to be considered when planning the clinical trial.

The ethical issues associated with the medical use of stem cells derived from fetal tissue have been debated in the literature and highlighted in the media for many decades [25, 26]. Several studies have looked at stakeholder views and found varying viewpoints. In 1994 Anderson et al [27] described a survey of 692 women, where 419 had never had a termination, that found the majority (94%) supported the use of fetal tissue in research. More recently a focus group study with 41 women, 10 of whom had never had a termination, found concerns about donating fetal tissue for research that included the potential for mishandling the terminated fetus in the laboratory and the idea that somehow the physical existence of the fetus would be reinstated if the stem cells were used in research [28]. For a small number of participants, the proposal to use MSCs that were fetal in origin raised ethical concerns. For many, however, this was not a barrier to the acceptability of SCT. Ultimately, information about SCT must fully disclose the fetal origin of the MSCs so that parents can make a decision in line with their own ethical and moral values.

## Limitations

Although the patient and parent group included individuals with a wide variety of experiences of life with OI, including differing OI severities and a range of ages, a key limitation of the study was that the majority of participants in this group were female, white British and well educated and this may limit generalisability. Another consideration for generalisability is that we did not interview any parents that had chosen to have a termination when OI was diagnosed during pregnancy. An important limitation was that all participants were self-selected and there may be responder bias towards people who hold strong pre-existing views regarding SCT. As such, care must be taken in interpreting the findings as they may not represent the wider population. Very few differences in the individual themes and sub themes that emerged were seen between the health professional, patient advocate and patient and parent groups, which may be explained by parents and patients having good medical knowledge about OI and that several participants in the patient and parent group were health professionals themselves. In the future it will be important to explore the experiences of parents who are offered SCT as part of the BOOSTB4 trial and speak to people who both accept and decline the invitation to participate.

## Conclusion

Key stakeholders support the application of a clinical trial of fetal MSC transplantation to treat severe OI administered during pregnancy or shortly after birth. Using plain language to explain SCT in a way that parents understand, clear expectation setting, substantial psychological support and time for reflection during the decision-making process will be crucial to allow parents to make informed decisions to take part in the BOOSTB4 clinical trial. Counselling and support needs may be different for families with a history of OI compared to families with a new and unexpected OI diagnosis.

## References

[1] Glorieux FH (2008). Osteogenesis imperfecta. Best Pract Res Clin Rheumatol.

[2] Forlino A, Marini JC (2016). Osteogenesis imperfecta. Lancet.

[3] Lim J, Grafe I, Alexander S, Lee B (2017). Genetic causes and mechanisms of Osteogenesis Imperfecta. Bone.

[4] Marini JC, Forlino A, Bachinger HP, Bishop NJ, Byers PH, Paepe A (2017). Osteogenesis imperfecta. Nat Rev Dis Prim.

[5] Sillence DO, Rimoin DL (1978). Classification of osteogenesis imperfecta. Lancet.

[6] Thomas IH, DiMeglio LA (2016). Advances in the Classification and Treatment of Osteogenesis Imperfecta. Curr Osteoporos Rep.

[7] Biggin A, Munns CF (2017). Long-Term Bisphosphonate Therapy in Osteogenesis Imperfecta. Curr Osteoporos Rep.

[8] Palomo T, Vilaca T, Lazaretti-Castro M (2017). Osteogenesis imperfecta: diagnosis and treatment. Curr Opin Endocrinol Diabetes Obes.

[9] Le Blanc K, Gotherstrom C, Ringden O, Hassan M, McMahon R, Horwitz E (2005). Fetal mesenchymal stem-cell engraftment in bone after in utero transplantation in a patient with severe osteogenesis imperfecta. Transplantation.

[10] Gotherstrom C, Westgren M, Shaw SW, Astrom E, Biswas A, Byers PH (2014). Pre- and postnatal transplantation of fetal mesenchymal stem cells in osteogenesis imperfecta: a two-center experience. Stem Cells Transl Med.

[11] Sagar R, Walther-Jallow L, David AL, Gotherstrom C, Westgren M (2018). Fetal mesenchymal stromal Cells: an opportunity for prenatal cellular therapy. Curr Stem Cell Rep.

[12] Braun V, Clarke V (2006). Using thematic analysis in psychology. Qual Res Psychol.

[13] Downey R, Geransar R (2008). Stem cell research, publics’ and stakeholder views. Health Law Rev.

[14] Dahan-Oliel N, Oliel S, Tsimicalis A, Montpetit K, Rauch F, Dogba MJ (2016). Quality of life in osteogenesis imperfecta: A mixed-methods systematic review. Am J Med Genet A.

[15] Tsimicalis A, Denis-Larocque G, Michalovic A, Lepage C, Williams K, Yao TR (2016). The psychosocial experience of individuals living with osteogenesis imperfecta: a mixed-methods systematic review. Qual Life Res.

[16] Wiggins S, Kreikemeier R (2017). Bisphosphonate therapy and osteogenesis imperfecta: The lived experience of children and their mothers. J Spec Pediatr Nurs.

[17] Hill CL, Baird WO, Walters SJ (2014). Quality of life in children and adolescents with Osteogenesis Imperfecta: a qualitative interview based study. Health Qual Life Outcomes.

[18] Santos MCD, Pires AF, Soares K, Barros L (2018). Family experience with osteogenesis imperfecta type 1: the most distressing situations. Disabil Rehabil.

[19] Dogba MJ, Rauch F, Tre G, Glorieux FH, Bedos C (2014). Shaping and managing the course of a child’s disease: parental experiences with osteogenesis imperfecta. Disabil Health J.

[20] Vanhelst J, Hardy L, Bert D, Duhem S, Coopman S, Libersa C (2013). Effect of child health status on parents’ allowing children to participate in pediatric research. BMC Med Ethics.

[21] Peay HL, Scharff H, Tibben A, Wilfond B, Bowie J, Johnson J (2016). “Watching time tick by…”: Decision making for Duchenne muscular dystrophy trials. Contemp Clin Trials.

[22] Sheppard M, Spencer RN, Ashcroft R, David AL (2016). Ethics and social acceptability of a proposed clinical trial using maternal gene therapy to treat severe early-onset fetal growth restriction. Ultrasound Obstet Gynecol.

[23] Smyth RM, Jacoby A, Elbourne D (2012). Deciding to join a perinatal randomised controlled trial: experiences and views of pregnant women enroled in the Magpie Trial. Midwifery.

[24] Oude Rengerink K, Logtenberg S, Hooft L, Bossuyt PM, Mol BW (2015). Pregnant womens’ concerns when invited to a randomized trial: a qualitative case control study. BMC Pregnancy Childbirth.

[25] Ishii T, Eto K (2014). Fetal stem cell transplantation: Past, present, and future. World J Stem Cells.

[26] Towns CR (2017). The science and ethics of cell-based therapies for Parkinson’s disease. Park Relat Disord.

[27] Anderson F, Glasier A, Ross J, Baird DT (1994). Attitudes of women to fetal tissue research. J Med Ethics.

[28] Pfeffer N (2008). What British women say matters to them about donating an aborted fetus to stem cell research: a focus group study. Soc Sci Med.

